# Identifying novel candidate compounds for therapeutic strategies in retinopathy of prematurity via computational drug-gene association analysis

**DOI:** 10.3389/fped.2023.1151239

**Published:** 2023-07-10

**Authors:** Edward F. Xie, Sarah Hilkert Rodriguez, Bingqing Xie, Mark D’Souza, Gonnah Reem, Dinanath Sulakhe, Dimitra Skondra

**Affiliations:** ^1^ Chicago Medical School, Rosalind Franklin University of Medicine and Science, Chicago, IL, United States; ^2^ Department of Ophthalmology and Visual Science, University of Chicago, Chicago, IL, United States; ^3^ Department of Medicine, University of Chicago, Chicago, IL, United States; ^4^ Center for Research Informatics, The University of Chicago, Chicago, IL, United States

**Keywords:** retinopathy of prematurity, bioinformatics & computational biology, bioinformatics, retina, drug discovery, network medicine

## Abstract

**Purpose:**

Retinopathy of prematurity (ROP) is the leading cause of preventable childhood blindness worldwide. Although interventions such as anti-VEGF and laser have high success rates in treating severe ROP, current treatment and preventative strategies still have their limitations. Thus, we aim to identify drugs and chemicals for ROP with comprehensive safety profiles and tolerability using a computational bioinformatics approach.

**Methods:**

We generated a list of genes associated with ROP to date by querying PubMed Gene which draws from animal models, human studies, and genomic studies in the NCBI database. Gene enrichment analysis was performed on the ROP gene list with the ToppGene program which draws from multiple drug-gene interaction databases to predict compounds with significant associations to the ROP gene list. Compounds with significant toxicities or without known clinical indications were filtered out from the final drug list.

**Results:**

The NCBI query identified 47 ROP genes with pharmacologic annotations present in ToppGene. Enrichment analysis revealed multiple drugs and chemical compounds related to the ROP gene list. The top ten most significant compounds associated with ROP include ascorbic acid, simvastatin, acetylcysteine, niacin, castor oil, penicillamine, curcumin, losartan, capsaicin, and metformin. Antioxidants, NSAIDs, antihypertensives, and anti-diabetics are the most common top drug classes derived from this analysis, and many of these compounds have potential to be readily repurposed for ROP as new prevention and treatment strategies.

**Conclusion:**

This bioinformatics analysis creates an unbiased approach for drug discovery by identifying compounds associated to the known genes and pathways of ROP. While predictions from bioinformatic studies require preclinical/clinical studies to validate their results, this technique could certainly guide future investigations for pathologies like ROP.

## Introduction

Retinopathy of prematurity (ROP) is a disorder characterized by the aberrant proliferation of retinal blood vessels within the incompletely vascularized retinas of premature infants (1, 2). Today, the pathophysiology of ROP is understood as a biphasic phenomenon initially manifesting as delayed retinal vasculature development in Phase 1 due to the relative hyperoxia of the immature retina (3). As the partially avascularized retina becomes increasingly metabolically active during perinatal development, the resulting relative hypoxia then triggers a compensatory secretion of angiogenic factors causing neovascularization in Phase II (4). While at times successful revascularization of the retina occurs, this condition can also progress to uncontrolled vasoproliferation into the vitreous which causes retinal scar formation or retinal detachment leading to permanent vision loss or blindness. Today, ROP is a leading cause of blindness in American children affecting 31.3% of all legally blind children treated at centers participating in the IRIS (Intelligent Research in Sight) Registry, a comprehensive clinical registry for eye disease in the US (5). As the survival rates of premature infants improved, ROP is also currently the leading cause of preventable childhood blindness worldwide, but its incidence is still expected to grow without future improvements in screening and treatment (1, 2, 6).

At present, the best preventative measure against ROP is preventing premature birth. Several targetable pathways for therapy have been identified with the oxygen-induced retinopathy (OIR) model, a hypoxia-driven angiogenesis model with similar mechanisms underlying the disease process of ROP, but the efficacy of some of these therapies have fallen short in clinical studies (7, 8). Decades of clinical trials have shown that while optimizing oxygen delivery during the perinatal period can reduce the risk of ROP development, there is still room for growth toward identifying a generalizable target oxygen saturation (SpO_2_) that balances infant mortality against ROP incidence (3). The Neonatal Oxygenation Prospective Meta-analysis (NeOProM) Collaboration observed in five randomized clinical trials that while a lower SpO_2_ target range reduces the risk of ROP in very preterm infants, it does so at the cost of increased risk of mortality (9). Alternative oxygen protocols have since arisen—notably a biphasic approach of adjusting oxygen saturations from a lower target range to a higher range as the infant matures, and one recent study noted decreased incidence and severity of ROP without elevated mortality using this method compared to conventional static oxygen standards (10).

Given the absence of a truly preventative therapy, the impetus falls on an experienced ophthalmologist to provide appropriate screening. Current evidence suggests that timely laser photocoagulation therapy or intravitreal anti-VEGF injections are effective therapies for preventing adverse retinal structural outcomes and improving visual outcomes (11, 12); however, these options unfortunately also have their own undesirable side effects (13). Laser photocoagulation reduces the risk of retinal detachment, but has also been associated with development of myopia (14–17). Additionally, there are notable concerns about the systemic absorption of intravitreal injections with anti-VEGF agents which have unknown effects on developing organs. Nevertheless, a randomized trial of intravitral injections with the anti-VEGF bevacizumab found no effects on neurodevelopmental scores at higher dosages (18), and ranibizumab in a separate trial was not associated with structural abnormalities (19) although ranibizumab notably has higher rates of reactivation than bevacizumab (20). One recent meta-analysis did however find a low risk of associated severe neurocognitive impairment associated with bevacizumab, but the current quality of evidence in the literature is limited (21). There is thus still a demand for novel therapies aimed at both prevention and treatment that may safely extend to all premature infants.

For a multifactorial pathology like ROP with numerous aberrant biological pathways at play, there may not be just one drug, one gene, one pathway, or one target. As even a single genomic variance can disrupt the protein-protein network, the interplay of multiple biochemical pathways should be studied. Systems medicine examines the interaction of distinct genetic and pharmacologic networks to identify novel targets for therapy. Previously, this method has identified potential drug targets for other complex pathologies including cancers, refractory epilepsy, Alzheimer's, and even other ocular conditions (22–28).

The creation of any novel drug for clinical applications is an expensive and time-consuming procedure, but this is a dilemma of drug discovery that network medicine can overcome by identifying already-approved pharmaceuticals and repurposing them for novel uses. By studying drugs and compounds with known safety profiles and pharmacokinetic/pharmacodynamics data, future clinical testing for an alternate indication is made less challenging as well. Prior studies on the OIR model have provided a wealth of knowledge on key pathway and genomic biomarkers to further the strength of this bioinformatics approach. We hypothesized that we could identify novel therapeutics for ROP using data on the existing genetic and proteomic biomarkers through this network-centric method.

## Methods

### Literature search and data extraction

We queried the NCBI database (https://www.ncbi.nlm.nih.gov/gene/) to identify genes with annotations for ROP (as of Dec 2022) based on previously published PubMed studies of ROP animal models, human studies, and genetic association studies. Only studies that demonstrated a significant association between ROP and their proposed pathways and genomic biomarkers were used, and articles which reported a negligible relationship were excluded in order to curb the frequency of false-positive findings. To make sure that the substance of chosen publications supported the conclusions, we carefully read through their entire texts. For this analysis, the genes that were found to be strongly related with ROP in the corresponding studies were used. Ethics approval was not necessary because neither humans nor animals were employed in this study.

### Discovering potential ROP therapeutic targets via enrichment analysis

To find possible pharmacological therapies for ROP, the degree of association between compounds and candidate ROP target genes/molecules was analyzed based on the theory that drugs with a greater association to corresponding disease genes will function with greater efficacy (29, 30). Compounds that strongly interact with the ROP genes found using gene enrichment analysis of multiple chemical-gene association databases could therefore possibly be used as therapeutic targets one day.

To perform gene set enrichment analysis (GSEA), we input the gene dataset from our PubMed query into the ToppFun function of the ToppGene Suite (http://toppgene.cchmc.org/). ToppGene aggregates 22,832 genes and a total of 77,146 drug annotations from 5 distinct databases including CTD, Drug Bank, Stitch, Broad Institute CMAP up, and Broad Institute CMAP down to produce a Pharmacome for predicting drug-gene interactions. ToppGene constructs a representative profile from the entered gene set and compares it to its annotated 22,832 test set genes in order to identify the drugs with the greatest associations. A hypergeometric distribution with the Bonferroni adjustment is used to assess statistical significance. Drugs with a false discovery rate (FDR) adjusted *P*-value of 0.05 were accounted for in this analysis.

### Selection of drugs/chemicals useful in ROP

The final list of compounds is filtered for chemicals like ethanol and asbestos which are associated to the ROP gene list but devoid of any known clinical indications as well as drugs like tacrolimus and cyclophosphamide which have clinical indications but have significant toxicities to human neonates. The final analysis also includes repeat drugs identified by the different databases used to construct the Pharmacome. The compound with the highest *P*-value amongst the repeats is preserved in the final analysis.

### Visualization of the networks

We used Cytoscape to visualize the connections between the enriched pharmaceutical drugs and the select list of ROP genes. The drug-gene network is captured using a prefuse-directed layout based on the edge betweenness centrality measure (31). In this graphical representation, gene nodes are displayed in green while the colors of drug nodes correlate to their respective drug-classes. This prefuse-directed layout uses closeness centrality to visualize hub nodes which are nodes with significantly more connections to other nodes. Closeness centrality is a measure of the average shortest distance to all other nodes and is represented by the size of the nodes as well as centrality. Thus, the largest nodes have the greatest closeness centrality and are the most interconnected points within the network. Only genes with three or more drug associations are visualized in the network.

The functional enrichment of the ROP genes also led to the creation of a gene-pathway network. Using the Lynx database system, enrichment analysis was performed for Gene Ontology (GO), disease, and pathway databases (32). The top ten most significant pathways from the enrichment study by *P*-value were selected for visualization along with their associated ROP genes to create a gene-pathway network. With a prefuse-directed layout based on edge betweenness and nodal size expressing proximity centrality, we visualized and analyzed the network once more using Cytoscape (31). In this network, the drug nodes are again colored green whereas the pathway nodes are colored blue.

## Results

We identified 51 unique genetic and proteomic markers associated with retinopathy of prematurity from prior human studies, animal models, genomic studies cited in PubMed. The ToppGene software recognized 47 genes out of the 51 as having significant annotations in the drug-gene-target databases of CTD, Stitch, Drug Bank, Broad Institute CMAP. Out of the 47 genes, 9 of these genes (Casp8, Atf4, Lgals1, Sema3A, Mir223, Malat1, TEAD4, Arg2, Sucnr1) were identified from animal studies of OIR models that noted a possible association to ROP. While there are no published genome-wide association studies (GWAS) for ROP to date, the remaining 38 genes are generated from human gene expression data. A review of literature revealed that 10 of the 38 genes (VEGFA, NOS3, IGF1, TNF, IL6, ACE, EPO, ANGPT1, C3, C5) associated to ROP are derived from studies of human vitreous and/or retinal tissue. Within the ToppGene drug-gene-target databases there are 77,146 annotated drugs and compounds, and our enrichment analysis of the select 47 genes identified 6,603 chemical compounds with an FDR adjusted *P*-value cutoff of 0.05. As this drug Pharmacome for ROP is generated from multiple drug databases utilized by ToppGene, there are a number of repeat chemicals depending on the database's drug-gene annotations. Additionally, there are a number of drugs with significant toxicities or compounds without any clinical indications that are in theory associated with genes related to ROP. Thus, we manually filtered out the redundant drugs and crafted a list of the top 50 significant compounds without deleterious effects to humans.

The most common class of drug within the top 50 significant compounds are antioxidants which guard against oxidative stress generated by reactive oxidants. This includes dietary micronutrients such as ascorbic acid, curcumin, and omega-3s as well as drugs with antioxidant effects such as N-acetylcysteine and penicillamine. We identified ascorbic acid (*P* = 7.76 × 10^−21^) or vitamin C, an antioxidant and essential nutrient in humans utilized as a cofactor for many enzymatic reactions, as the compound with the strongest association to ROP genes by significance. Ascorbic acid is functionally associated with 21 of the 47 disease causal genes which is the greatest number of genes out of all potential compounds. Additional antioxidants include polyphenol micronutrients including curcumin (*P* = 3.74 × 10^−14^), the biologically active ingredient in turmeric, which affects 18 of the 47 ROP genes and apigenin (*P* = 3.60 × 10^−10^), a compound found in chamomile plants, celery, and parsley, which affects 9 of the 47 ROP genes. Niacin (*P* = 4.25 × 10^−14^), otherwise known as vitamin B3, and alpha-lipoid acid, an omega-3 fatty acid, were also highly significant to ROP genes associating with 11 and 10 genes, respectively. Drugs approved for medical use with anti-oxidative effects include N-acetylcysteine (*P* = 1.60 × 10^−17^), an antidote for acetaminophen overdose via antioxidant formation, and penicillamine (*P* = 2.04 × 10^−14^), a chelating agent.

Other top drug classes currently used for indications approved by the US Food and Drug Administration (FDA) include anti-diabetics, non-steroidal anti-inflammatory drugs (NSAIDs), and cardiovascular drugs. Top compounds from the anti-diabetics class include metformin (*P* = 4.30 × 10^−14^), which is the most commonly prescribed drug for type II diabetes and affects 14 of the 47 ROP genes, and thiazolidinediones such as troglitazone (*P* = 4.94 × 10^−10^), rosiglitazone (*P* = 7.58 × 10^−10^) and pioglitazone (*P* = 3.75 × 10^−9^) which activate peroxisome proliferator-activated receptors (PPAR) for various effects and affect 17, 18, and 8 of the ROP genes, respectively. NSAIDs include ibuprofen (*P* = 6.13 × 10^−11^), diclofenac (*P* = 1.27 × 10^−9^), celecoxib (*P* = 4.11 × 10^−9^), and aspirin (*P* = 8.88 × 10^−9^) which associate with 12, 12, 9, and 12 of the ROP genes. Cardiovascular drugs identified in the top 50 most significant compounds include nifedipine and hydralazine which are calcium channel blockers (CCBs), losartan and telmisartan which are angiotensin II receptor blockers (ARBs), and enalapril and quinapril which are angiotensin-converting enzyme (ACE) inhibitors.

We also separately identified possible clinical compounds associated to the 38 genes distinguished from human studies. The top 10 compounds associated to human ROP genes include vitamin C, simvastatin, enalapril, curcumin, vitamin B3, acetylcysteine, penicillamine, nifedipine, phenylephrine, and metformin. As human genes compromised 80% of the total ROP biomarkers identified from the NCBI database, this independent enrichment analysis using only the human genes yielded largely the same top drug classes and compounds as the comprehensive list in Table 1.

**Table 1:** Top 50 filtered drugs targeting ROP genes predicted by toppGene database in order of *P*-value.

Filtered Position	Unfiltered Position	Name	Source	*P*-value	q-value FDR B&H	Hit Count in Query List	Hit Count in Genome
1	1	Ascorbic Acid	CTD	7.76 × 10^−21^	1.30 × 10^−16^	21	626
2	4	Simvastatin	CTD	1.44 × 10^−18^	5.79 × 10^−15^	19	581
3	7	Acetylcysteine	CTD	1.60 × 10^−17^	3.83 × 10^−14^	20	780
4	13	Niacin	CTD	4.24 × 10^−15^	5.37 × 10^−12^	11	139
5	14	Ricinelaidic acid	Stitch	4.48 × 10^−15^	5.37 × 10^−12^	10	95
6	17	Penicillamine	CTD	2.04 × 10^−14^	2.01 × 10^−11^	10	110
7	18	Curcumin	CTD	2.74 × 10^−^14	2.41 × 10^−^11	18	850
8	19	Losartan	CTD	2.88 × 10^−14^	2.41 × 10^−11^	11	165
9	20	Capsaicin	CTD	3.00 × 10^−14^	2.41 × 10^−11^	15	488
10	22	Metformin	CTD	4.30 × 10^−14^	3.28 × 10^−11^	14	400
11	27	Enalapril	CTD	1.21 × 10^−13^	7.53 × 10^−11^	10	131
12	36	Thioctic Acid	CTD	1.04 × 10^−12^	4.84 × 10^−10^	10	162
13	37	Furosemide	CTD	1.17 × 10^−12^	5.23 × 10^−10^	10	164
14	43	U0126	Stitch	2.55 × 10^−12^	9.96 × 10^−10^	13	430
15	44	Genistein	Stitch	2.80 × 10^−12^	1.07 × 10^−9^	18	1,117
16	49	Betamethasone-d5	Stitch	5.35 × 10^−12^	1.81 × 10^−9^	19	1,340
17	57	Minocycline	CTD	1.00 × 10^−11^	2.94 × 10^−9^	8	88
18	61	Phenylephrine	CTD	1.91 × 10^−11^	5.25 × 10^−9^	13	505
19	63	Telmisartan	CTD	2.22 × 10^−11^	5.91 × 10^−9^	8	97
20	77	Melatonin	CTD	5.84 × 10^−11^	1.27 × 10^−8^	10	243
21	78	Ibuprofen	CTD	6.13 × 10^−11^	1.32 × 10^−8^	12	437
22	79	Nifedipine	CTD	7.14 × 10^−11^	1.51 × 10^−8^	8	112
23	81	Glutathione	CTD	7.33 × 10^−11^	1.52 × 10^−8^	11	339
24	87	Valsartan	CTD	1.16 × 10^−10^	2.24 × 10^−8^	8	119
25	91	hydralazine	Stitch	1.39 × 10^−10^	2.54 × 10^−8^	9	186
26	92	Atorvastatin Calcium	CTD	1.39 × 10^−10^	2.54 × 10^−8^	9	186
27	94	Triamcinolone	Stitch	1.47 × 10^−10^	2.60 × 10^−8^	7	73
28	102	Dantrolene	CTD	2.37 × 10^−10^	3.88 × 10^−8^	6	41
29	105	quinapril	CTD	2.53 × 10^−10^	4.04 × 10^−8^	5	18
30	111	Apigenin	CTD	3.60 × 10^−10^	5.45 × 10^−8^	9	207
31	117	troglitazone	CTD	4.94 × 10^−10^	7.08 × 10^−8^	17	1,329
32	120	LMWH	Stitch	5.45 × 10^−10^	7.58 × 10^−8^	13	663
33	133	rosiglitazone	CTD	7.58 × 10^−10^	9.56 × 10^−8^	18	1,571
34	138	Isoproterenol	CTD	9.70 × 10^−10^	1.18 × 10^−7^	15	1,015
35	144	Diclofenac	CTD	1.27 × 10^−9^	1.48 × 10^−7^	12	570
36	146	candesartan	Stitch	1.37 × 10^−9^	1.58 × 10^−7^	8	162
37	157	Rutin	CTD	1.96 × 10^−9^	2.09 × 10^−7^	7	105
38	161	Propolis	CTD	2.56 × 10^−9^	2.66 × 10^−7^	6	60
39	164	Etiocobalamin	Stitch	2.77 × 10^−9^	2.82 × 10^−7^	8	177
40	165	Nitroprusside	Stitch	2.78 × 10^−9^	2.82 × 10^−7^	9	261
41	166	Amlodipine	CTD	2.83 × 10^−9^	2.86 × 10^−7^	6	61
42	178	Pioglitazone	CTD	3.75 × 10^−9^	3.52 × 10^−7^	8	184
43	180	Sulforafan	CTD	3.87 × 10^−9^	3.60 × 10^−7^	11	494
44	187	Deferoxamine	CTD	4.09 × 10^−9^	3.66 × 10^−7^	8	186
45	188	Celecoxib	Stitch	4.11 × 10^−9^	3.66 × 10^−7^	9	273
46	191	Fenofibrate	Stitch	4.52 × 10^−9^	3.97 × 10^−7^	9	276
47	198	Galangin	CTD	5.53 × 10^−9^	4.68 × 10^−7^	6	68
48	208	Tempol	CTD	7.20 × 10^−9^	5.80 × 10^−7^	6	71
49	215	Aspirin	CTD	8.88 × 10^−9^	6.93 × 10^−7^	12	678
50	216	Nitroglycerin	Stitch	9.11 × 10^−9^	7.07 × 10^−7^	8	206

**Figure 1:**
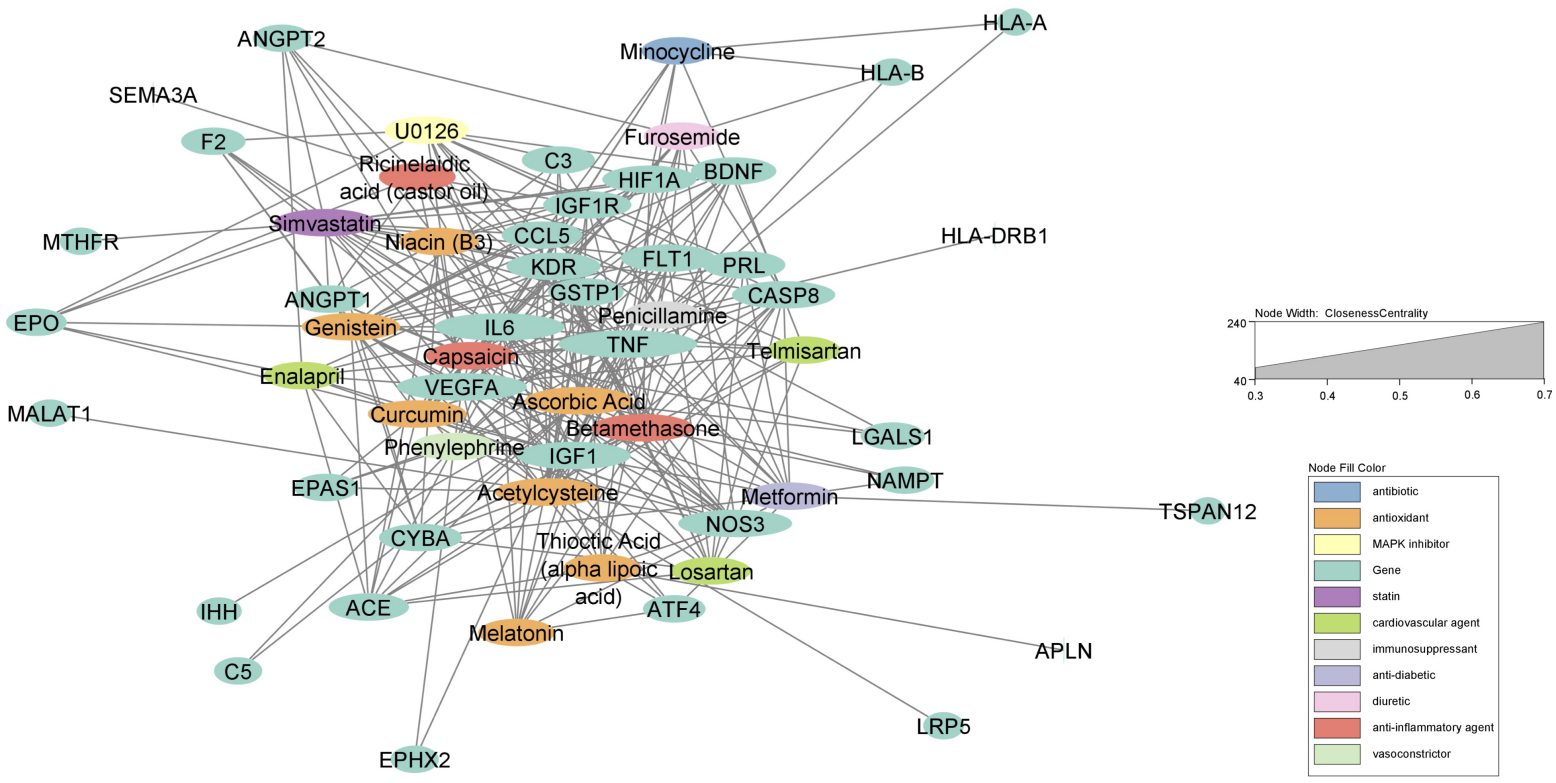
Force-directed graph of drug-gene interactions. Node size and edges are represented based on centrality metrics analysis. Drugs are in red, and genes are green with respect to color.

Out of the 47 biomarkers deemed significant to ROP, there were several genes considered “hub” genes which means they were nodes in the network with a particularly high degree of connection to the top compounds identified from the analysis. These key genes with high closeness and betweenness centrality in the drug-gene interaction network include TNF, VEGFA, IL6, NOS3, IGF1, CASP8, HIF1A, KDR, and FLT1 (Figure 1). These hub genes are most associated with “vasculature development” which refers to the creation of vessels from endothelial precursors, “response to decreased oxygen levels”, and “angiogenesis” per their associated GO terms for biological processes (Table 2). The GO terms “vascular development” and “angiogenesis” are independently identified in which the former refers to the creation of vessels from ehdothelial precursors while the latter refers to the formation of new vessels from existing vessels.

**Table 2:** Most significant gene ontology description of genes associated with genes related to ROP.

ID	Name	*P*-value	q-value FDR B&H	Hit Count in Query List	Hit Count in Genome
GO:0001944	vasculature development	1.46 × 10^−25^	5.62 × 10^−22^	30	1,239
GO:0036293	response to hypoxia	9.97 × 10^−23^	1.92 × 10^−19^	22	547
GO:0035239	tube morphogenesis	4.52 × 10^−22^	3.48 × 10^−19^	29	1,467
GO:0035295	tube development	1.38 × 10^−21^	8.87 × 10^−19^	31	1,880
GO:0001525	angiogenesis	4.62 × 10^−21^	2.22 × 10^−18^	24	873
GO:0072359	circulatory system development	6.15 × 10^−21^	2.63 × 10^−18^	31	1,977
GO:0051240	regulation of multicellular organismal process	7.32 × 10^−20^	2.82 × 10^−17^	30	1,950
GO:0014070	response to organic cyclic compound	3.88 × 10^−17^	9.97 × 10^−15^	26	1,628
GO:0048660	regulation of smooth muscle cell proliferation	4.63 × 10^−17^	1.12 × 10^−14^	14	223
GO:0033002	muscle cell proliferation	4.45 × 10^−16^	9.51 × 10^−14^	15	334

**Figure 2:**
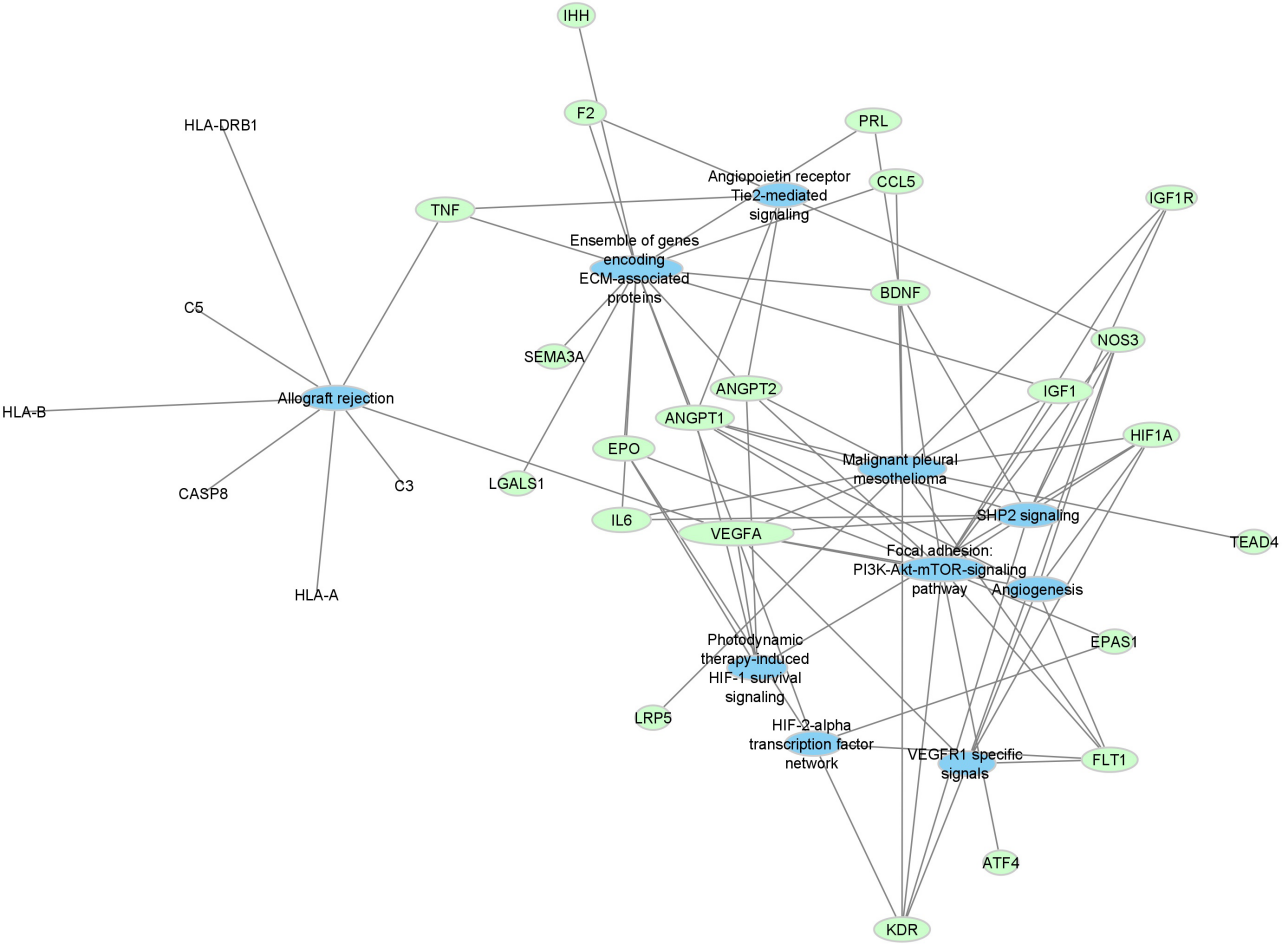
Force-directed graph of drug-gene interactions. Node size and edges are represented based on centrality metrics analysis. Pathways are in blue, and genes are green with respect to color.

Top pathways associated with the development of ROP using the Lynx Enrichment tool include “HIF-1 signaling pathway,” “PI3K-Akt signaling pathway,” “angiogenesis,” “allograft rejection,” “SHP2 signaling,” and “Signaling events mediated by VEGFR1 and VEGFR2” (Figure 2). Within the drug-pathway network, the genes which hold the highest degree value include IL6, TNF, VEGFA, CASP8, IGFR1, IGF1, and KDR. The pathway with the highest closeness centrality and greatest degree of gene connections is “PI3K-Akt signaling pathway, “ and the top pathway by significance is “HIF-1 signaling pathway.” These pathways are associated with the aforementioned 7 top genes in addition to 8 others including EPO, FLT1, HIF1A, ANGPT1, ANGPT2, NOS3, PRL, and BDNF. These 15 genes comprise the principal targets for the top drugs found in our ROP Pharmacome (Figure 1). Gene ontology terms associated with these top 15 genes include “response to hypoxia”, “regulation of blood vessel endothelial cell migration”, and “vasculature development” (Table 2).

The complete list of biomarkers, compounds, and gene-drug correlation are available in Supplementary S1 which will be publicly available once the manuscript is accepted for publication.

## Discussion

Screening and management for ROP continues to evolve as new technologies for imaging, machine learning, and high-throughput analyses emerge. The determination of biomarkers is one key avenue of exploration that can advance our understanding of ROP development, disease progression, and possible targets for therapy (33). One possible approach for identifying potential biomarkers utilizes network medicine—an integration of distinct biological networks like protein-protein interactions, metabolic pathways, and gene regulatory networks to understand the mechanisms of disease and to discern novel targets and candidate compounds for investigation. As biological networks are closely entangled, even a single pathological disruption can elicit a cascading effect to alter an otherwise carefully balanced system. Norrie's disease and familial exudative vitreoretinopathy (FEVR) are retinal conditions, which present similarly to ROP engendered, engendered by a single genetic variant many of which overlap with ROP including NDP, FZD4 TSPAN12, and LRP5 from our analysis. Thus, a network medicine approach can identify the tie-ins between multiple pathways to gain an understanding of larger biological relationships that otherwise wouldn't be understood by conventional reductionist approaches of “one drug-one target-one gene.” In this study, we take a network medicine approach to clarify the relationships between the known genes of ROP and current pharmacologic compounds to predict medical drugs targets.

For decades, oxidative stress has been hypothesized to play a major role in the pathogenesis of ROP (34, 35). As neonates, particularly premature neonates, lack appropriate levels of antioxidants to eliminate ROS, they are especially susceptible to oxidative stress and sensitive to ROS-activated signaling pathways when transferred from a hypoxic intrauterine condition to a normoxic or even hyperoxic environment with supplemental oxygen at birth (36, 37). Thus, antioxidants as preventative therapies have long been viewed as promising agents that can mitigate the damage from oxidative stress. Vitamin E, vitamin C, zeazanthin, ubiquinone (CoQ10), and alpha lipoid acid, an omega-3 fatty acid, are all potent antioxidants identified from our analysis that also been tested in both animals and humans; however, the promising results from animal models for these antioxidants often failed to translate in human studies (33, 38). Ascorbic acid (AscA) or vitamin C, the most significant compound in our analysis, has been linked to ROP and retinal angiogenesis as plasma AscA levels were lower in premature infants who developed ROP (39). Unfortunately, there is no evidence that AscA supplementation has any significant benefits for protecting against ROP in premature infants (40). Additionally, other antioxidant therapies like zeaxanthin and omega-3 fatty acids supplementation have also shown equivocal results in preventing ROP clinically, and vitamin E trials have largely been abandoned due to increased risks of neonatal morbidities like sepsis and intraventricular hemorrhage (41–44).

Curcumin and N-acecylcysteine (NAC) are two major antioxidants identified amongst the most significant compounds in our analysis. While they exhibit anti-oxidative effects, they have also been shown to suppress pathological angiogenesis by inhibiting expression of angiogenic factors and/or apoptotic factors like NOS3, VEGF, IL-6, IGF-1, and TNF which are hub genes in our analysis that encode for factors that are normally elevated in the serum and vitreous of ROP infants (45–50). As recent studies have identified VEGF as one of, if not the most significant, angiogenic factors responsible for ROP (34), VEGF inhibitors are the principle agents of therapy despite potential drawbacks (12, 51). One recent study demonstrated that curcumin administered during hypoxia can reduce retinal neovascularization and VEGF mRNA expression in oxygen-induced retinopathy mice in a dose-dependent manner, and *in vitro* experiments showed that curcumin decreased VEGF protein and mRNA levels similar in degree to ranibizumab, an anti-VEGF drug used to treat ROP (52, 53). Of note, neither curcumin nor NAC have adverse effects in infants (54, 55), and both already have strong evidence in preclinical studies towards protecting against retinal disorders (56, 57). Although curcumin exhibits poor bioavailability which limited human studies in the past, novel formulations like Longvida curcumin have arisen that accumulate in the retina and are currently being trialed in retinal conditions like AMD (58). Meanwhile, NAC has already shown promise for retinitis pigmentosa, another retinal disorder related to oxidative stress, as a phase 1 clinical trial in adults demonstrated improved macular cone function and reduced oxidative damage with oral NAC therapy (59). While NAC has never been studied in the context of ROP models, NAC treatment in diabetic rat models showed decreased levels of ROS contents in the retina and also attenuated VEGF expression in retinal blood vessels and signs of retinopathy under microscopy (60). Both curcumin and NAC are promising candidates to be studied to repurpose as ROP therapy thanks to their safety in infants as well as anti-oxidative and anti-angiogenic effects, but further studies will be necessary in the future.

Our study also identified several anti-diabetics including metformin and various thiazolidinediones (TZD). TZDs have well-documented anti-angiogenic properties via their PPAR-y agonism which induces suppression of VEGF expression in the retina as seen *in vitro* and *in vivo* studies (61), and retinal vascular tissues are one of the few sites where PPAR-y receptors are abundantly expressed (62). Furthermore, rosiglitazone, wnfhich we identified amongst the top 50 most significant compounds, has been shown to delay the onset of proliferative diabetic retinopathy possibly due to its anti-angiogenic activity in a review of longitudinal medical records (63). Metformin has similarly exhibited anti-angiogenic effects in mouse oxygen-induced retinopathy (OIR) models by reducing the expression of VEGF receptors (64). Due to an array of neuroprotective, anti-angiogenic, and anti-proliferative effects, metformin has been identified in various preclinical and clinical studies as a drug with strong repurposable potential for neurodegenerative disorders (65), various cancers like glioblastoma (66, 67), and also retinal pathologies like AMD (68). Studies have also suggested that metformin exhibits anti-inflammatory effects by reducing the production of NO, prostaglandins, and pro-inflammatory cytokines (IL-6, TNF-a) which are normally elevated in ROP via inhibition of NF-KB (69, 70). Today, metformin is widely prescribed for pregnant patients with polycystic ovarian syndrome and gestational diabetes mellitus with proven safety and tolerability (71–73), but neonatal outcomes of metformin use in pregnancy remain unclear (74, 75). While metformin is untested in children under the age of 2, its safety will be studied in infants affected by hypoxic ischemic encephalopathy (76).

Common cardiovascular agents including ACE inhibitors and ARBs are top candidates in our analysis. The renin-angiotensin system (RAS) mediates the homeostatic control of fluid balance, tissue perfusion, and arterial pressure. Today, ACE inhibitors and ARBs are used to target different steps in this system to control blood pressure and improve heart and kidney function. Recent studies have reported on the existence of an ocular renin-angiotensin system as evidence points to the expression of RAS components—renin, angiotensinogen, and angiotensin-converting enzyme (ACE)—in the eye of humans and animals with RAS playing a possible role in the early stages of vascularization in rats (76–78). One study found a multifold increase in the expression of RAS components including ACE and HIF-1a—hub genes identified from our analysis—within the human vitreous of ROP patients and the retina of OIR rats (79). As RAS is induced by hypoxia, there is a possibility that pharmacological intervention of RAS could also arrest or reduce the progression of ROP development. Lisinopril and telmisartan which were both identified in our analysis blocked the up-regulation of VEGF and HIF-1a in the retina of OIR rat pups as much as bevacizumab, an anti-VEGF agent, and suppressed retinal functional changes on ERG (80). Interestingly, ACE inhibitors have been suggested to attenuate erythropoietin (EPO) activity, a key biomarker that is elevated in the vitreous of stage 4 ROP patients (81, 82). While ACE inhibitors are commonly used for pediatric cardiology patients, they are contraindicated in pregnancy and should be considered with caution, especially for preterm infants (83). One recent study observed Enalapril-use in both preterm and term infants without structural heart disease in the neonatal intensive care unit and found that the most common side effects include hyperkalemia (13%), elevated serum creatinine (5%), and hypotension (4%); nonetheless, there may be still potential for repurposing these drugs in ROP therapeutics (84).

Inflammation has long played a key role in ROP pathogenesis and has always been considered an important pharmacologic target. Of note, cyclooxygenase (COX)-2 seems to play a hand in neovascularization via the generation of prostaglandin E2 and activation of prostaglandin EP3 receptors (85). Inhibition of COX-2 in the retina also decreased neovascularization in an ROP mice model by 37% (86). Indeed, an early preliminary report of ophthalmic ketorolac reduced the risk of severe ROP in preterm neonates without adverse effects (87). Since then NSAIDs have been tested with other promising therapies like caffeine, which was also identified in our study but outside the top 50 candidates, in order to target both oxidative stress and inflammation with notable success in OIR models (88). Larger studies are certainly still necessary, however, as the effectiveness of prophylactic NSAIDs and caffeine still requires further validation from future clinical studies (89).

There are inherited retinal conditions including NDP and FEVR which manifest similarly to ROP with abnormal angiogenesis and incomplete vascularization of the retina (90). The drug-gene Pharmacome constructed for ROP in this study suggests that there are several overlapping key genes associated with not only FEVR and Norrie's disease but also with ROP including NDP, FZD4, TSPAN12, and LRP5 (90–94). Unfortunately, these shared genes represent only a small subset of the overall drug-gene network for ROP and lack significant associations with the top drugs identified in this study. Similarly, the primary biological pathway—the WNT signaling pathway—associated to both FEVR and Norrie's disease was not identified in this study as a targetable pathological network to ROP despite the presence of key genes affecting WNT signaling in our drug-gene Pharmacome (95). While it's unlikely that the drugs we've discussed relate significantly to Norrie's disease or FEVR based on the current evidence, these are pathologies that may similarly benefit from our network medicine methodology in drug discovery as our understanding of their genetic basis grows.

### Limitations

While this study attempts to create a drug-gene network to identify pharmacological targets for ROP, the model is still far from perfect. ROP pathogenesis is a growing field in need of novel, predictive biomarkers, and our study utilizes only the known genomic and proteomic biomarkers derived in part from animal models that may not adequately translate to preterm humans. Because genomics-based predictions are built upon pre-existing research to pinpoint drug-gene interactions, a predicted computational model for rare, ocular pathologies like ROP is not going to be as robust when compared to more prevalent pathologies like cancer or diabetes because there's less data and identified biomarkers. Similarly, less commonly used drugs and more niche compounds have less toxicogenomics data. Thus, the more ubiquitously prescribed drugs may appear over-represented in enrichment analyses due to a greater number of known drug-gene interactions. Another constraint is that we did not account for the specific impact of the genes as either a protective or risk variant on gene progression; we gave equal value and emphasis on all genes identified from our NCBI query. Finally, there are intrinsic issues in databases like CTD and Drug Bank which partially rely on algorithms to make predictive drug-gene or gene-disease inferences that may contain false-positives. Ultimately, this research design mimics a meta-analysis of earlier studies, and it is necessary to take into consideration variations in standards of practice and experimental design amongst different studies.

## Conclusion

Our investigation into biomarkers of ROP uncovered many potential pharmacological therapies for this disorder. We identified several compounds untested for ROP such as NAC and curcumin that have known safety and tolerability profiles in preterm infants and are therefore prime candidates for repurposing. Other top drugs within our analysis include anti-diabetics like metformin and TZDs as well as cardiovascular agents like ARBs and ACE inhibitors which are FDA-approved for various indications but have unclear or unknown side effects in neonates. Of course, there is also a slew of other compounds including various NSAIDs and antioxidant nutrients which traditionally have been tested as ROP prophylactic therapy, and hopefully will continue to be tested with larger clinical studies. For a complex, multifactorial disease like ROP, multiple drug classes may be necessary to synergistically combat ROP's intricate pathogenesis. Thus, combination therapies are also valuable options to explore in future studies. While there are still drawbacks to using a bioinformatics approach, this model of utilizing open-source algorithms and databases as a method of investigation undoubtedly gives an unbiased insight into drug discovery. As our understanding of ROP risk factors and biomarkers grows with the explosion of new, innovative technologies and artificial intelligence, the accuracy of using a systems biology approach to predict drug-target-relationships will only improve. We believe that this computational system biology study can provide direction for future preclinical and clinical studies to elucidate the relationships between ROP and the significant drugs we've identified, and we are hopeful that this approach of using bioinformatics to investigate drug discovery can apply to other irremediable pathologies in the future as well.

## Data Availability

The original contributions presented in the study are included in the article/**Supplementary Material**, further inquiries can be directed to the corresponding author/s.
